# Toll-like receptor 2 agonists inhibit human fibrocyte differentiation

**DOI:** 10.1186/1755-1536-3-23

**Published:** 2010-11-24

**Authors:** Anu S Maharjan, Darrell Pilling, Richard H Gomer

**Affiliations:** 1 Department of Biochemistry and Cell Biology, MS-140, Rice University, 6100 S. Main Street, Houston, TX 77005-1892, USA; 2 Department of Biology, MS-3258, Texas A&M University, College Station, TX 77843-3258, USA

## Abstract

**Background:**

In healing wounds, some monocytes enter the wound and differentiate into fibroblast-like cells called fibrocytes. Since Toll-like receptors (TLRs) are present on monocytes, and pathogens that can infect a wound have and/or release TLR agonists, we examined whether TLR agonists affect fibrocyte differentiation.

**Results:**

When human peripheral blood mononuclear cells (PBMCs) were cultured with TLR3, TLR4, TLR5, TLR7, TLR8 or TLR9 agonists, there was no significant effect on fibrocyte differentiation, even though enhanced extracellular tumor necrosis factor (TNF)-α accumulation and/or increased cell surface CD86 or major histocompatibility complex (MHC) class II levels were observed. However, all TLR2 agonists tested inhibited fibrocyte differentiation without any significant effect on cell survival. Adding TLR2 agonists to purified monocytes had no effect on fibrocyte differentiation. However, some TLR2 agonists caused PBMCs to secrete a factor that inhibits the differentiation of purified monocytes into fibrocytes. This factor is not interferon (IFN)-α, IFN-γ, interleukin (IL)-12, aggregated immunoglobulin G (IgG) or serum amyloid P (SAP), factors known to inhibit fibrocyte differentiation. TLR2 agonist-treated PBMCs secrete low levels of IL-6, TNF-α, IFN-γ, granulocyte colony-stimulating factor and tumor growth factor β1, but combinations of these factors had no effect on fibrocyte differentiation from purified monocytes.

**Conclusions:**

Our results indicate that TLR2 agonists indirectly inhibit fibrocyte differentiation and that, for some TLR2 agonists, this inhibition involves other cell types in the PBMC population secreting an unknown factor that inhibits fibrocyte differentiation. Together, these data suggest that the presence of some bacterial signals can inhibit fibrocyte differentiation and may thus slow wound closure.

## Background

Following injury, circulating peripheral blood cells such as neutrophils, monocytes, dendritic cells and lymphocytes leave the bloodstream and enter the injured site. Once monocytes are in the injured site, they can differentiate into fibroblast-like cells called fibrocytes [1-8]. Fibrocytes have a distinct spindle-shaped appearance. Fibrocytes express hematopoietic markers, including CD45, major histocompatibility complex (MHC) class II, and CD34, along with stromal markers including collagen I, collagen III, and fibronectin [1,9,10]. Fibrocytes help to rebuild injured tissue by secreting angiogenic and fibrogenic growth factors as well as matrix metalloproteinases [11]. Fibrocytes are also contractile cells, which further helps to close wounds by pulling the wound edges together [4].

We found that the serum protein serum amyloid P (SAP) can directly inhibit monocytes from differentiating into fibrocytes [5]. Purified SAP inhibits fibrocyte differentiation, but other serum proteins such as serum amyloid A and C-reactive protein (CRP) are unable to inhibit the differentiation of monocytes into fibrocytes [5,12]. The profibrotic cytokines interleukin (IL)-4 and IL-13 directly activate monocytes to differentiate into fibrocytes, while cross-linked immunoglobulin G (IgG) and the proinflammatory cytokine interferon (IFN)-γ directly inhibit the differentiation of monocytes into fibrocytes [6,7]. Another proinflammatory cytokine, IL-12, activates some cells in the peripheral blood mononuclear cell (PBMC) population, possibly natural killer (NK) cells, to indirectly inhibit fibrocyte differentiation [7]. In human PBMC culture, IFN-α2b inhibits fibrocyte differentiation, but whether this acts directly on monocytes is unknown [13]. Other regulators of fibrocytes include the adenosine A_2A _receptor and cysteinyl leukotriene receptor 1 (CysLT1) [14,15]. The adenosine A_2A _receptor regulates cell proliferation and cytokine production, and blocking this receptor inhibits the recruitment of fibrocytes in bleomycin-treated mouse skin [14]. CysLT1 is a receptor for lipid mediators which promote fibroblast proliferation, fibroblast chemotaxis and collagen synthesis [15]. In mice with fluorescein isothiocyanate (FITC)-induced lung fibrosis, blocking CysLT1 inhibits the appearance of fibrocytes [15].

It is unclear why some of the above factors affect fibrocyte differentiation. However, we hypothesized that SAP prevents fibrocyte differentiation in the circulation and that aggregated IgGs prevent fibrocyte differentiation because aggregated IgGs signify the presence of infection. We hypothesized that after monocytes detect an infected wound, monocytes do not differentiate into fibrocytes, since closing an infected wound could cause further damage such as gangrene, an infectious wound closure that results in further decay of the surrounding cells [16,17]. The immune system can also recognize pathogens using Toll-like receptors (TLRs) [18-24]. TLR agonists include pathogen-specific molecules such as lipopolysaccharides (LPSs) from gram-negative bacteria, lipotechoic acid (LTA) from gram-positive bacteria, flagellin from bacteria, single-stranded DNA (ssDNA) from viruses and unmethylated DNA from bacteria [24,25]. TLR signaling pathways trigger innate immune responses through nuclear factor (NF)-κB-dependent and IFN-regulatory factor-dependent pathways [19]. Since TLRs are present on monocytes, we examined whether TLR agonists could also affect the differentiation of monocytes into fibrocytes.

## Methods

### Culturing PBMC with TLR agonists or IFN-α

Blood was collected from healthy adult volunteers in accordance with specific approval of Rice University's Institutional Review Board. Written consent was received from all volunteers, and all samples were deidentified before analysis. PBMCs were isolated and incubated in serum-free media (SFM) as described previously [5-7,26]. TLR agonists (Invivogen, San Diego, CA, USA) were reconstituted in endotoxin-free water (Invivogen). All experiments were done with at least three different batches of agonists. IFN-α was obtained from EMD-Calbiochem (Darmstadt, Germany). A dilution series of TLR agonists (or the same volume of water as a control) was made in serum-free medium. A quantity of 100 μl of serum-free medium, TLR agonist or IFN-α dilution, or water dilution, was added to duplicate wells of a 96-well tissue culture plate (BD Biosciences, San Jose, CA, USA). A quantity of 100 μl of human PBMCs at a concentration of 5 × 10^5 ^cells/ml in serum-free medium was then added to each well. On day 5, fields of PBMCs were photographed using a phase-contrast microscope, and the number of cells per image was counted. Cells were then fixed and stained, and fibrocytes were counted as previously described [5-7,26].

### Preparation of monocytes

Monocytes were purified from 5 × 10^7 ^PBMCs using an EasySep Monocyte Depletion Kit (catalog no. 19059; StemCell Technology, Vancouver, BC, Canada) according to the manufacturer's instructions. To determine the purity of the monocytes, cells were analyzed using flow cytometry (FACScan, BD Biosciences; or Accuri C6 cytometer, Accuri Cytometers Inc., Ann Arbor, MI, USA) as described previously [7]. A sample of each monocyte preparation was stained with 5 μg/ml primary antibodies against the T cell marker CD3, the monocyte marker CD14, the NK cell marker CD16, the B cell marker CD19 and the leukocyte marker CD45 as previously described [7]. Monocytes obtained were greater than 95% pure as determined by the expression of CD14. Monocytes were 99% CD45-positive, 0.93% CD3-positive, 0.93% CD16-positive and 1% CD19-positive. To assess the effect of TLR2 agonists on monocytes, 5 μl of TLR2 agonist or water were added to 245 μl of serum-free medium. A quantity of 100 μl of serum-free medium, TLR2 agonist dilution or water dilution was added to a well of a 96-well plate, with each condition represented in duplicate wells. A quantity of 100 μl of purified human monocytes at a concentration of 5 × 10^5 ^cells/ml in serum-free medium was then added to each well. Fibrocytes were fixed, stained and counted after monocytes were cultured for 5 days.

### Treating monocytes with conditioned media from PBMC stimulated with TLR2 agonists

To make conditioned medium, a dilution of TLR2 agonists was made as described above. A quantity of 100 μl of either SFM, the TLR2 agonist dilution or the corresponding water dilution was added to duplicate wells of a 96-well plate along with 100 μl of PBMCs at a concentration of 5 × 10^5 ^cells/ml in SFM. After 3 days of incubation, 180 μl of the conditioned media from one well representing each condition were transferred into an Eppendorf tube, snap-frozen in liquid nitrogen and stored at -20°C. For the well from which the medium was not removed, at day 5 the fibrocytes were fixed, stained and counted as previously described [5-7]. Also on day 5, monocytes were prepared from the same donor as described above. A quantity of 50 μl of monocytes at a concentration of 1 × 10^6 ^cells/ml was incubated with 50 μl of the day 3 conditioned medium. After 5 days, cells were fixed and stained, and the number of fibrocytes was counted.

### Detection of cytokines and SAP by enzyme-linked immunosorbent assay

The day 3 conditioned media were analyzed for IFN-α, IFN-γ and IL-12 using enzyme-linked immunosorbent assay (ELISA) according to the manufacturer's instructions (Peprotech, Rocky Hill, NJ, USA). The day 3 conditioned media were also analyzed for IL-2, IL-4, IL-5, IL-6, IL-10, IL-12, IL-13, IL-17A, IFN-γ, TNF-α, granulocyte colony-stimulating factor (G-CSF) and tumor growth factor (TGF)-β1 using a multi-analyte profiler ELISArray kit according to the manufacturer's instructions (SABiosciences, Frederick, MD, USA).

The day 3 conditioned media were also analyzed for SAP as described previously [5,27], with the exception that the ELISA plates were coated overnight at 4°C with mouse antihuman SAP antibody (SAP-5; Sigma, St. Louis, MO, USA) diluted 1:1,000 in phosphate-buffered saline (PBS) instead of 50 mM sodium carbonate buffer, and undiluted day 3 conditioned media were assayed.

### Staining PBMC with CD86 or MHC Class II

Human PBMCs were cultured in the presence or absence of 8.9 μg/mL TLR3 agonist Poly (I:C), 0.89 μg/mL TLR7 agonist imiquimod (IMIQ), or 2.0 μg/ml nucleotide oligomerization domain (NOD)-like receptor (NLR) agonist peptidoglycan (PGN) in 1 ml in the well of a 48-well plate. On day 1 or 3, 900 μl of the conditioned media were carefully pipetted out and transferred into an Eppendorf tube, snap-frozen in liquid nitrogen and stored at -20°C. A quantity of 500 μl of ice-cold 50 mM ethylenediaminetetraacetic acid (EDTA) in PBS was then added to the PBMCs for 5 minutes at 4°C. The cells were vigorously resuspended with a plastic transfer pipette. The cells were transferred into an Eppendorf tube, collected by centrifugation at 300 × *g *for 5 min and the supernatant was discarded. The remaining cells on the plates were washed with 1 ml of ice-cold PBS, and this solution was added to the first pellet of cells. The cells were again collected by centrifugation at 300 × *g *for 5 min and then resuspended in 200 μl of 4% bovine serum albumin (BSA) in PBS. Cells treated with TLR agonists were divided into two separate Eppendorf tubes. The cells were incubated with 5 μg/ml antihuman CD86 or 5 μg/ml antihuman Human leukocyte antigen-DR/DP/DQ (HLA-DR/DP/DQ) (MHC class II) (both from BD Biosciences) for 30 min at 4°C as described previously [28]. Meanwhile, untreated cells were divided into three separate Eppendorf tubes. These cells were incubated with 5 μg/ml antihuman CD86 or 5 μg/ml antihuman HLA-DR/DP/DQ, or they were kept in 4% BSA in PBS for 30 min at 4°C. All of the cells were washed three times in 1 ml of ice-cold PBS and then incubated with 2.5 μg/ml goat antimouse FITC (Southern Biotechnology, Birmingham, AL, USA) for 30 min at 4°C as described previously [28]. After washing the cells three times in 1 ml of ice-cold PBS, the cells were resuspended in 100 μl of 4% BSA-PBS, and the staining was analyzed using flow cytometry with an Accuri C6 cytometer (Accuri Cytometers Inc.).

### Detection of Ig molecules by Western blot analysis

Human IgG (Jackson ImmunoResearch Laboratories, West Grove, PA, USA) was diluted to 10 μg/ml, 1 μg/ml and 0.1 μg/ml in PBS. A quantity of 10 μl of conditioned media from day 3 or diluted human IgG was mixed with 2.5 μl of sodium dodecyl sulfate (SDS) sample buffer containing 20 mM dithiothreitol (DTT) and heated to 100°C for 5 min. After electrophoresis of the samples on 4-15% Tris-glycine polyacrylamide gels (Bio-Rad Laboratories, Hercules, CA, USA), proteins were transferred to polyvinylidene difluoride (PVDF) membranes (Millipore, Bedford, MA, USA) in Tris-NaCl-SDS buffer containing 20% methanol. Western blot staining was performed as described previously [5], with the exception that the detection antibody was 0.05 μg/ml biotinylated goat Fab'(2) antihuman Ig (H+L) (Southern Biotechnology) followed by 1:5,000 ExtrAvidin-Peroxidase staining (Sigma).

### Treating monocytes with different combinations of cytokines

After analyzing the day 3 conditioned media with multi-analyte ELISArray kits, we calculated the concentrations of IL-6, IFN-γ, TNF-α, G-CSF, and TGF-β1 in LTA (TLR2 agonist)-treated conditioned media, LPS (TLR4 agonist)-treated conditioned media and control conditioned media. Cocktails of cytokines corresponding to twice the observed concentrations were made in SFM. A quantity of 100 μl of purified human monocytes at a concentration of 5 × 10^5 ^cells/ml were incubated in 100 μl of SFM, 100 μl of the above cytokine cocktails or 100 μl of the cytokine cocktail with all five of the above cytokines at either 2,000 pg/ml, 1,000 pg/ml, 200 pg/ml or 20 pg/ml. After 5 days, cells were fixed and stained, and fibrocytes were counted.

### Statistics

Statistical analysis was performed using GraphPad Prism software (GraphPad Software, San Diego, CA, USA). Statistical significance was determined using either analysis of variance (ANOVA) or a *t*-test, and significance was defined as *P *< 0.05.

## Results

### TLR3, TLR4, TLR5, TLR7, TLR8 and TLR9 agonists do not inhibit the differentiation of PBMCs to fibrocytes

To investigate the role of TLR agonists on fibrocyte differentiation, human PBMCs were cultured in the presence of various TLR agonists. Since immune cells can affect each other, we used PBMCs instead of purified monocytes to more closely mimic a human immune system. All of the TLR agonists were reconstituted in endotoxin-free water, and a control series of water dilutions had no discernible effect on fibrocyte differentiation (data not shown).

**Figure 1:**
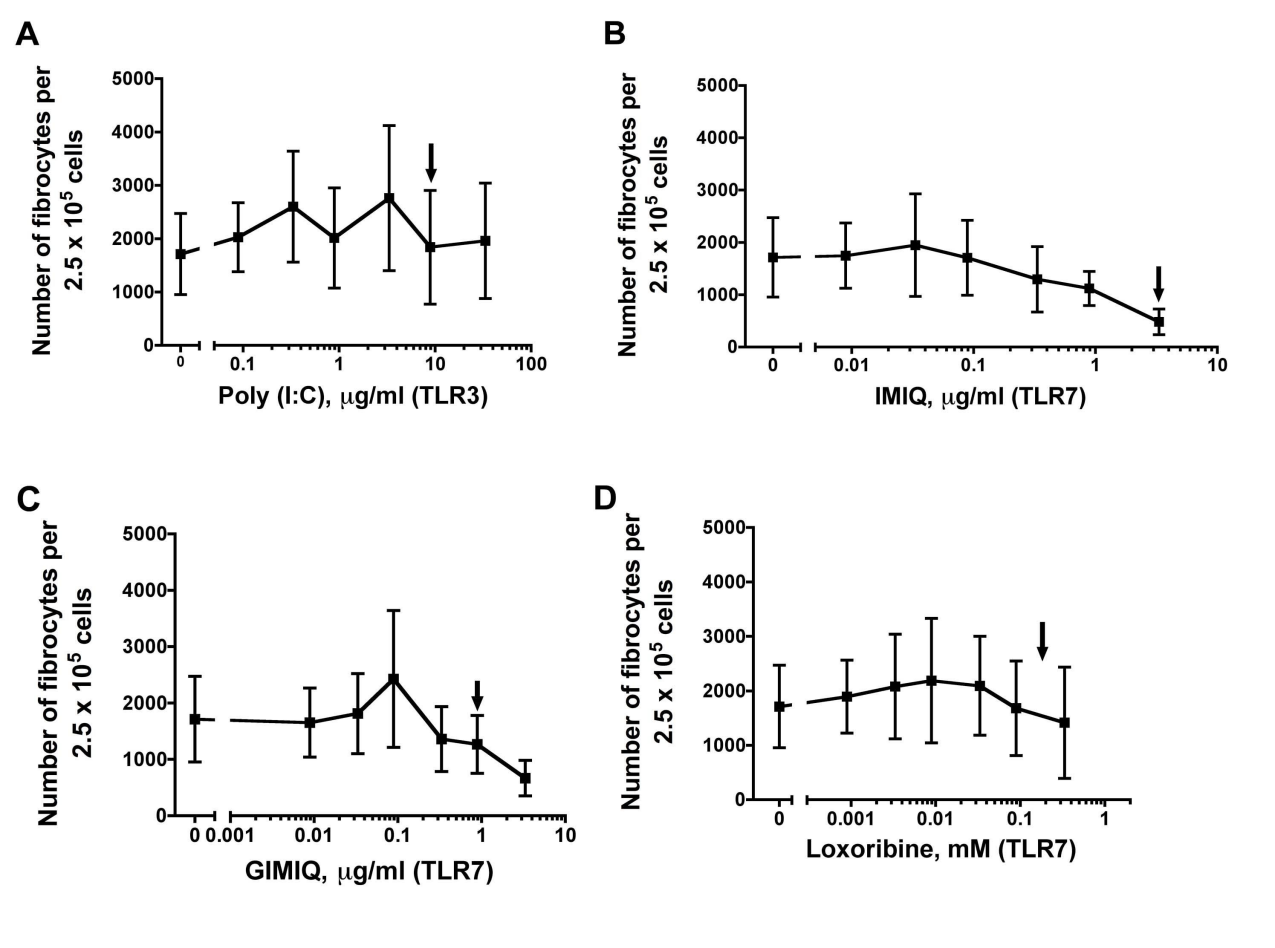
**Toll-like receptor 3 (TLR3) and TLR7 agonists do not affect fibrocyte differentiation**. Human peripheral blood mononuclear cells (PBMCs) were cultured in different concentrations of the TLR3 agonist polyinosine-polycytidylic acid (Poly I:C) and the TLR7 agonists imiquimod (IMIQ), gardiquimod (GIMIQ) and loxoribine. After 5 days, the cells were air-dried, fixed and stained, and the number of fibrocytes was counted. The results are means ± SEM of fibrocytes per 2.5 × 10^5 ^PBMCs (n = 3 separate experiments). Although high concentrations of IMIQ and GIMIQ appear to decrease the number of fibrocytes compared to no addition, the decrease is not significant by either analysis of variance (ANOVA) or *t*-test. Arrows indicate the reported concentrations for the TLR agonists previously shown to be functional (Table 1).

**Figure 2:**
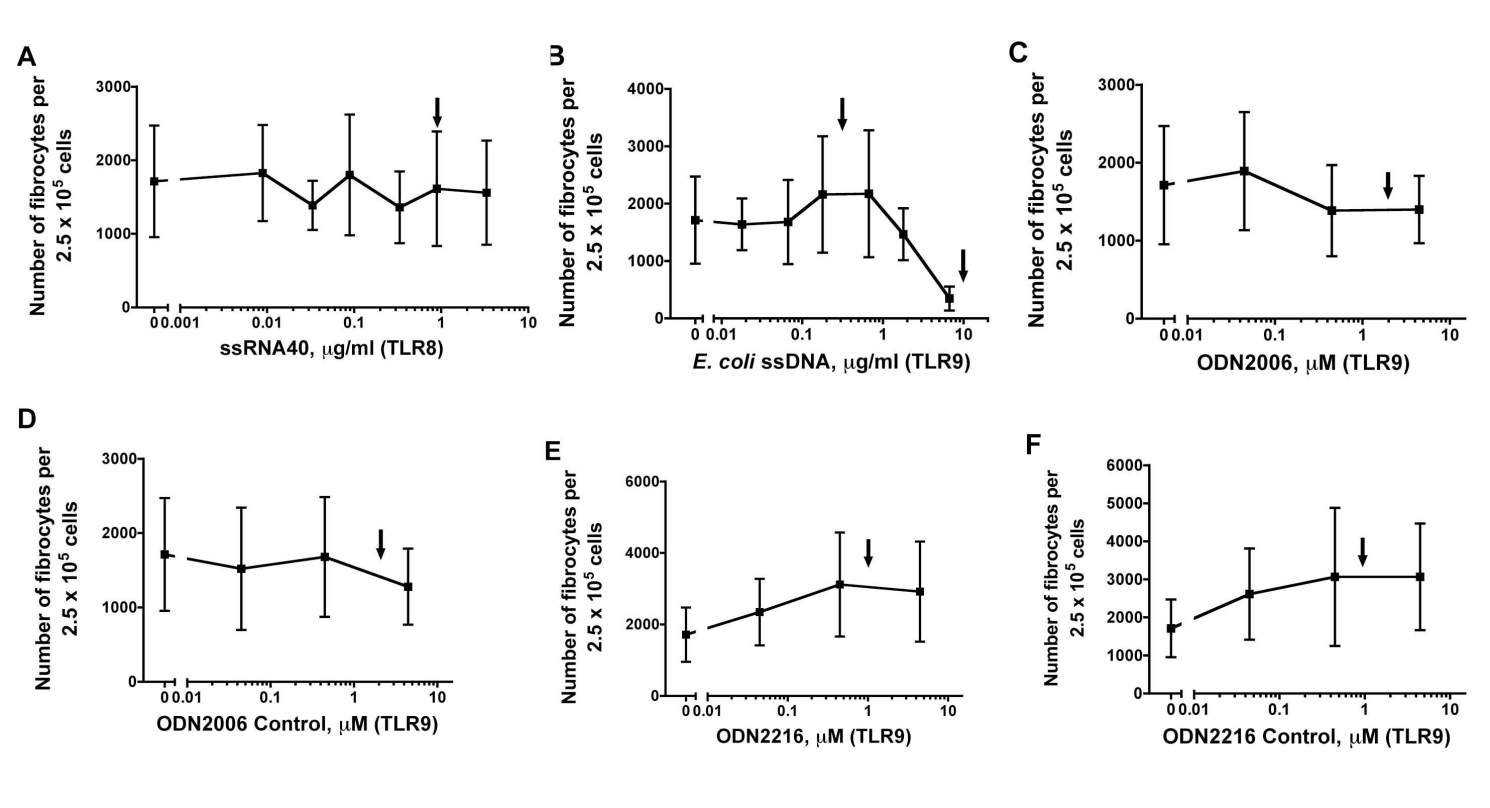
**TLR8 and TLR9 agonists do not affect fibrocyte differentiation**. Human PBMCs were cultured in different concentrations of the TLR8 agonist single-stranded RNA40 (ssRNA40) and the TLR9 agonists *Escherichia coli *ssDNA, ODN2006 and ODN2216. ODN2006 and ODN2216 are synthetic oligonucleotides that contain CpG dinucleotides at specific sequences. The controls for these oligonucleotides are ODN2006 control and ODN2216 control, which contain GpC dinucleotides instead of CpG dinucleotides. After 5 days, the cells were air-dried, fixed and stained, and the number of fibrocytes was counted. The results are means ± SEM of fibrocytes per 2.5 × 10^5 ^PBMCs (n = 3 separate experiments). Although high concentrations of *E. coli *ssDNA appear to decrease the number of fibrocytes compared to no addition, the decrease is not significant by either ANOVA or *t*-test. High concentrations of ODN2216 and ODN2216 control appear to increase fibrocyte number compared to no addition, but the increase is not significant by either ANOVA or *t*-test. Arrows indicate the concentrations of TLR agonists used by other workers (Table 1).

**Figure 3:**
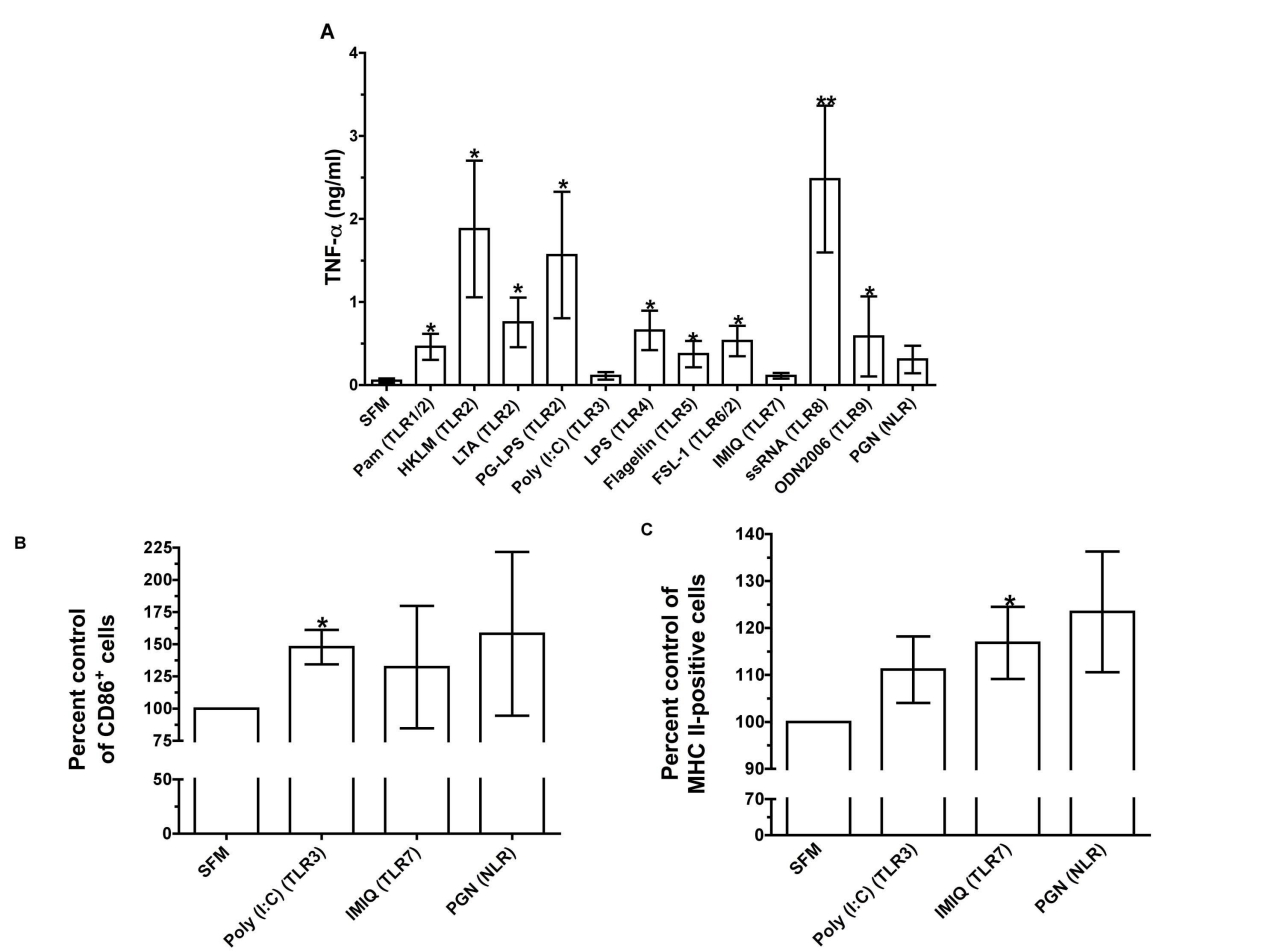
**TLR agonists affect PBMCs**. **(A)**. Human PBMCs were cultured in the absence (serum-free medium (SFM) control) or the presence of 0.89 μg/ml TLR2 agonists Pam3CSK4, 8.9 × 10^7 ^cells/ml heat-killed *Listeria monocytogenes *(HKLM), 1.79 μg/ml lipotechoic acid (LTA), or 8.9 μg/ml lipopolysaccharides from *Porphyromonas gingivalis *(PG-LPS), 8.9 μg/ml TLR3 agonist Poly (I:C), 0.89 μg/ml TLR4 agonist LPS, 0.89 μg/ml TLR5 agonist flagellin, 0.89 μg/ml TLR2/6 agonist FSL-1, 0.89 μg/ml TLR7 agonist IMIQ, 0.89 μg/ml TLR8 agonist ssRNA, 4.47 μM TLR9 agonist ODN 2006 or 1.79 μg/ml nucleotide oligomerization domain (NOD)-like receptor (NLR) agonist peptidoglycan (PGN). On day 3, the conditioned media were tested for the presence of tumor necrosis factor (TNF)-α using enzyme-linked immunosorbent assay. The results are means ± SEM of TNF-α concentration (n = 3 or more separate experiments). (**B) **Human PBMCs were cultured in the presence or absence of Poly (I:C), IMIQ and PGN as described for Figure 3A. After 1 day, PBMCs were removed and stained for CD86 (n = 3 separate experiments). **(C) **Human PBMCs were cultured in the presence or absence of 8.9 μg/ml TLR3 agonist Poly (I:C), 0.89 μg/ml TLR7 agonist IMIQ or 1.79 μg/ml NLR agonist PGN. On day 3, PBMCs were removed from the tissue culture plate and stained for major histocompatibility complex (MHC) class II. The results are means ± SEM of positive MHC class II cells (n = 4 separate experiments). *P < 0.05 and **P < 0.01 compared to no-agonist control (SFM) according to *t*-test.

All TLR agonists were used in dose ranges that had induced responses in previous studies (Table 1). TLR3, TLR7, TLR8 and TLR9 agonists consisted of viral, bacterial or synthetic nucleic acids that stimulated their corresponding receptors. When added to PBMCs, none of these agonists showed any significant effect on fibrocyte differentiation (Figures 1 and 2). The TLR9 agonist *Escherichia coli *ssDNA appears to decrease fibrocyte differentiation at 10 μg/ml (Figure 2B), but it was not significant by either ANOVA or *t*-test. One batch of the TLR7 agonist IMIQ inhibited fibrocyte differentiation at 3.3 μg/ml, but other batches of IMIQ from the same manufacturer had no significant effect on fibrocyte differentiation. Although TLR3, TLR7, TLR8 and TLR9 agonists do not affect fibrocyte differentiation, the TLR8 agonist ssRNA and the TLR9 agonist ODN2006 that we used for these studies induced extracellular TNF-α accumulation by PBMCs (Figure 3A). TLR3 and TLR7 agonists do not significantly enhance the production of TNF-α, but the TLR3 agonist Poly (I:C) significantly increased the number of CD86-positive cells at day 1 (Figure 3B) and the TLR7 agonist IMIQ significantly increased the number of HLA-DR/P/Q (MHC class II)-positive cells at day 3 (Figure 3C), which suggest that these agonists still have biological effects on PBMCs.

**Table 1:** TLR agonists stimulate different cytokines and effectors in various cell types^a^

Agonist	TLR	Concentration used	Cell type	Effect	References
Pam3CSK4 (synthetic)	TLR2	0.30 μg/ml	PBMC	Induction of IL-6	[38]
Lipomannan *M. smegmatis *(LM-MS)	TLR2	10 μg/ml	PBMC	Cellular aggregation	[48]
Heat-killed *Listeria monocytogenes *(HKLM)	TLR2	3 × 10^7 ^cells/ml	PBMC	Induction of type I and type II IFN	[49]
Lipotechoic acid from *S. aureus *(LTA)	TLR2	10 μg/ml	RAW264.7	Increased expression of IL-1β, TNF-α, IL-6, and IP-10	[50]
Lipopolysaccharide from *P. gingivalis *(PG-LPS)	TLR2	0.1 μg/ml	U373	Secretion of IL-6	[51]
FSL-1 (synthetic)	TLR2	0.1 μg/ml	PBMC	Induction of Type I and Type II IFN	[49]
Poly (I:C) (synthetic)	TLR3	10 μg/ml	PBMC	Production of IL-8, MCP-1, and TNF-α	[52]
*E. coli *K12 lipopolysaccharide (LPS)	TLR4	0.01, 0.1, and 1 μg/ml	PBMC	Production of TNF-α, IL-6, and IL-10	[30,31]
*S. typhimurium *flagellin	TLR5	0.1 μg/ml	PBMC	Activation of NF-κB	[29]
Imiquimod (IMIQ) (synthetic)	TLR7	3 μg/ml	PBMC	Expression of IFN, TNF-α, IL-6, and IL-8 increased	[53]
Gardiquimod (GIMIQ) (synthetic)	TLR7	1 μg/ml	Plasmacytoid dendritic cells	Expression of IFN-α, IFN-β, and RANTES increased	[54]
Loxoribine (synthetic)	TLR7	0.2 mM	RAW264.7 mouse macrophage cell line NK cells	Expression of IL-23 p19 increased Activation of NK cells	[55,56]
ssRNA40 (synthetic)	TLR8	1 μg/ml	Peripheral blood	Activation of TNF-α	[57]
*E. coli *ssDNA/LyoVec	TLR9	0.3-10 μg/ml	RAW264.7	Production of nitrite	[58]
ODN2006 (synthetic)	TLR9	2 μM	PBMC	Increased production of IFN-γ, IFN-α, IL-6, IL-8, and IL-12	[59,60]
ODN2216 (synthetic)	TLR-9	1 μM	Plasmacytoid dendritic cells	Decreased production of IFN-α	[61]
Peptidoglycan from *S. aureus *(PGN)	NLR	0.1-10 μg/ml	HEK-293	Activation of NF-κB	[62]

^a^This table summarizes the concentration of agonists used by other workers. TLR, Toll-like receptor; PBMC, human peripheral blood mononuclear cells; IL, interleukin; IFN, interferon; TNF, tumor necrosis factor; NF-κB, nuclear factor-κB; HEK-293, human embryonic kidney-293 cells; PGN, peptidoglycan.

**Figure 4:**
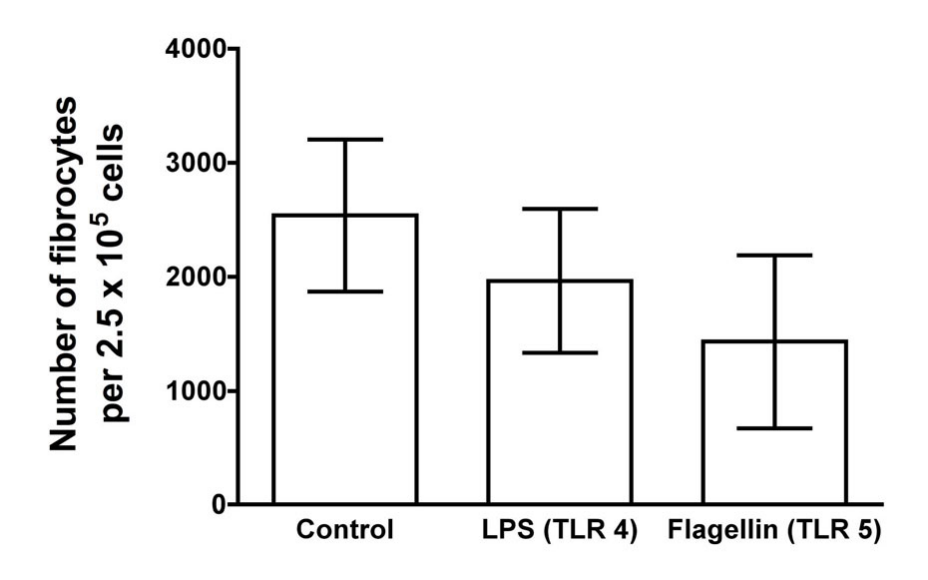
**TLR4 and TLR5 agonists do not affect fibrocyte differentiation**. Human PBMCs were cultured in the presence or absence of 0.89 μg/ml TLR4 agonist LPS or 0.89 μg/ml TLR5 agonist flagellin. After 5 days, the cells were air-dried, fixed and stained, and the number of fibrocytes was counted. The results are means ± SEM of fibrocytes per 2.5 × 10^5 ^PBMCs (n = 6 separate experiments). Although LPS and flagellin appear to decrease the number of fibrocytes compared to no addition, the decrease is not significant by either ANOVA or *t*-test.

TLR4 recognizes bacterial LPSs, and TLR5 recognizes bacterial flagellin [29-31]. We observed that 0.89 μg/ml LPS or 0.89 μg/ml flagellin had no effect on fibrocyte differentiation (Figure 4). Although the number of fibrocytes decreased in PBMCs treated with LPS or flagellin, *t*-tests showed no significant difference compared to no TLR agonist. Both LPS and flagellin caused PBMCs to increase their accumulation of extracellular TNF-α (Figure 3A), which suggests that the LPS and flagellin we used have biological effects on PBMCs. Together, the data suggest that TLR3, TLR4, TLR5, TLR7, TLR8 and TLR9 agonists do not significantly affect the *in vitro *differentiation of human fibrocytes.

### TLR2 agonists inhibit the differentiation of PBMCs to fibrocytes

**Figure 5:**
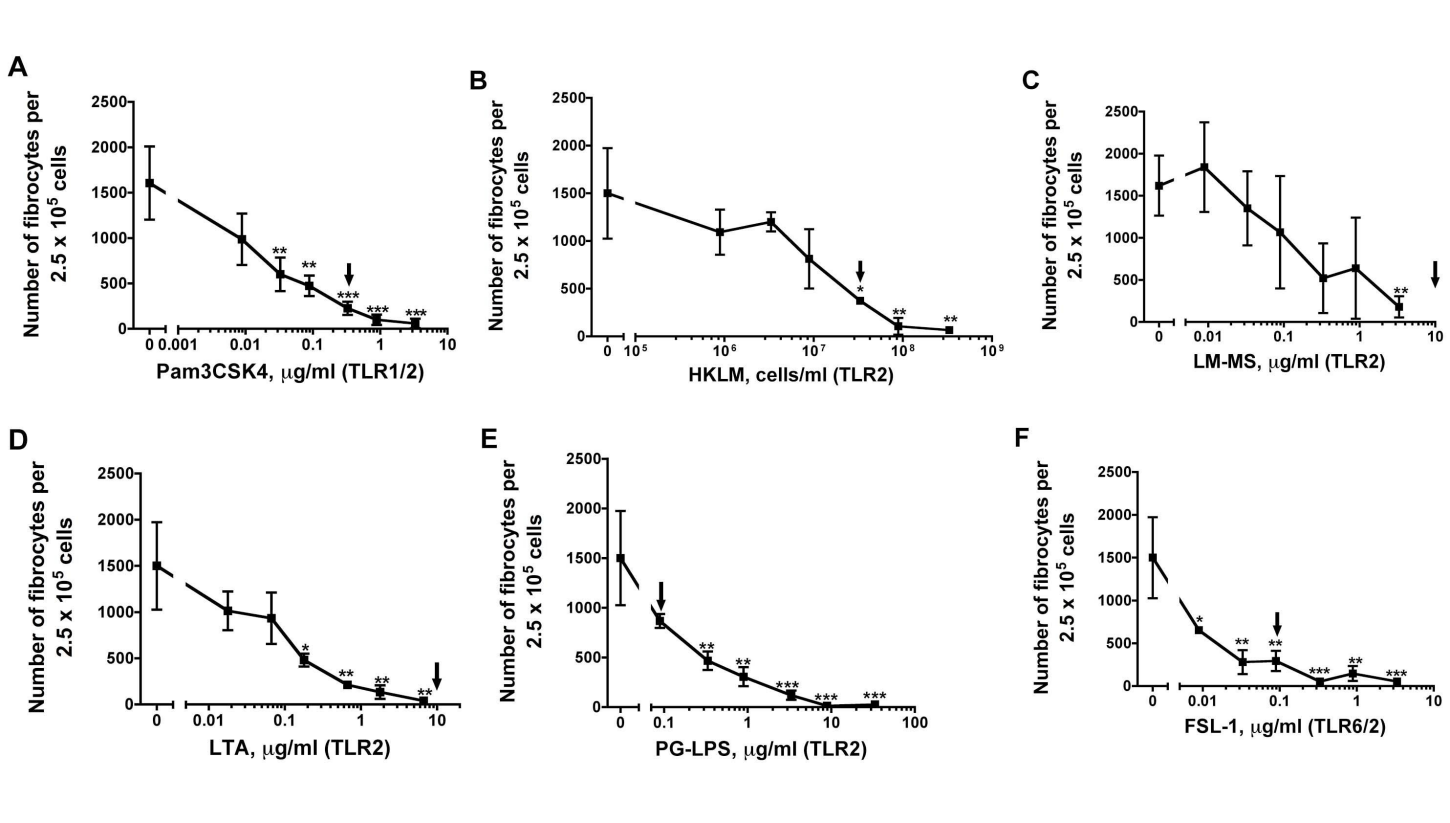
**TLR2 agonists inhibit fibrocyte differentiation**. Human PBMCs were cultured in different concentrations of the TLR2 agonists Pam3CSK4, HKLM, lipomannans from *Mycobacterium smegmatis *(LM-MS), LTA, PG-LPS and FSL-1. After 5 days, the cells were air-dried, fixed and stained, and the number of fibrocytes was counted. The results are means ± SEM of fibrocytes per 2.5 × 10^5 ^PBMCs (n = 6 separate experiments for Pam3CSK4 and n = 3 separate experiments for the rest of the TLR2 agonists). The absence of error bars indicates that the error was smaller than the plot symbol. Statistical significance was analyzed by ANOVA using Dunnett's test to compare values against the no-agonist control for all the agonists except LM-MS, which was analyzed by *t*-test. *P < 0.05, **P < 0.01, and ***P < 0.001 compared to no-agonist control. Arrows indicate the concentration of TLR2 agonists used by other workers (Table 1).

**Figure 6:**
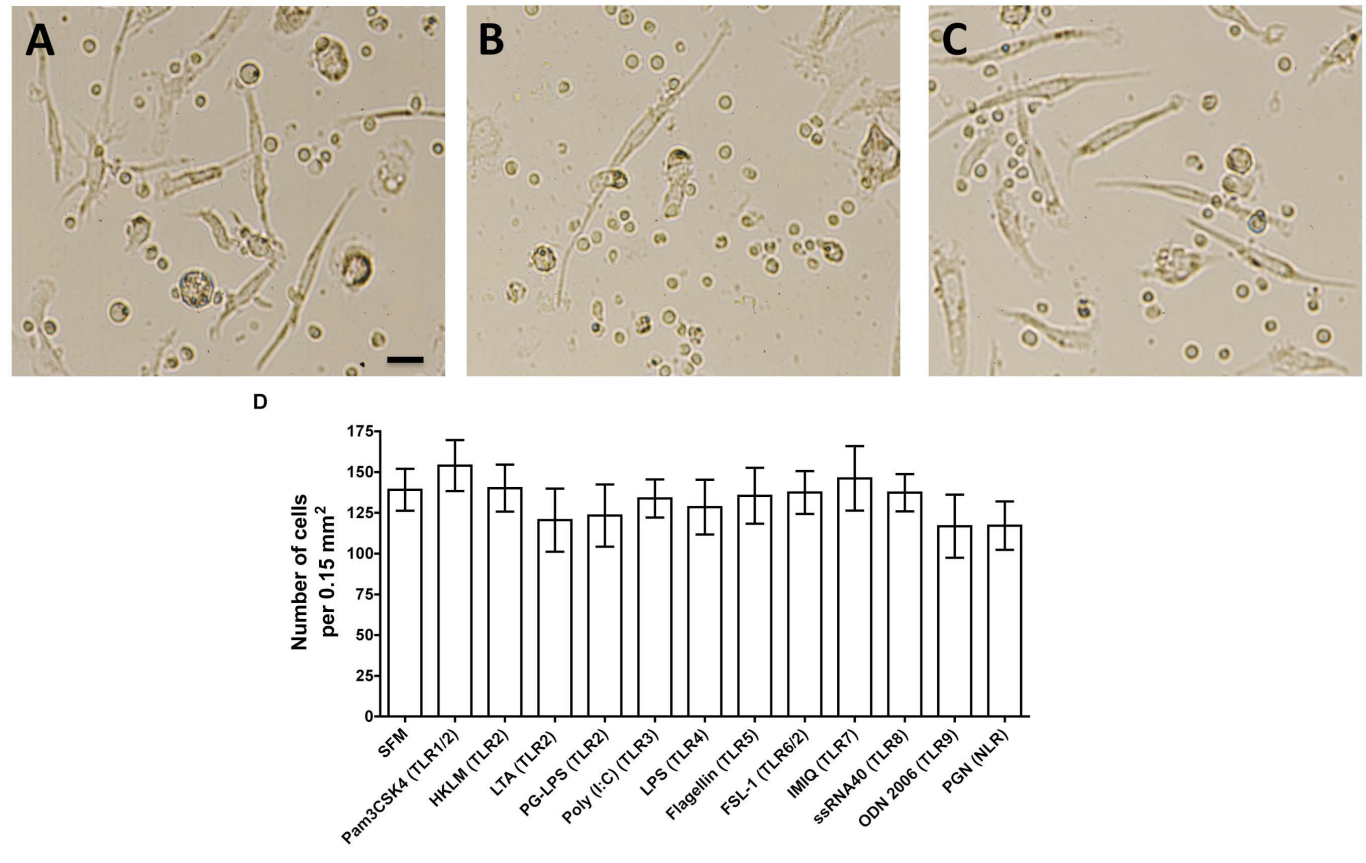
**Human PBMCs cultured in the presence of TLR2 agonists are viable**. Human PBMCs were cultured in the **(A)**. absence or **(B) **presence of 1.79 μg/ml TLR2 agonist LTA or **(C) **0.89 μg/ml TLR7 agonist IMIQ. After 5 days, the cells cultured in different conditions were photographed. Bar is 20 μm. (**D) **Human PBMCs were cultured in the absence (SFM control) or presence of 0.89 μg/ml TLR2 agonists Pam3CSK4, 8.9 × 10^7 ^cells/ml HKLM, 0.89 μg/ml LM-MS, 1.79 μg/ml LTA, 8.9 μg/ml PG-LPS or 0.89 μg/ml FSL-1 for 5 days. After 5 days, the cells were photographed and the number of cells per picture was counted. The results are means ± SEM of cells per 0.15 mm^2 ^(n = 3 separate experiments).

TLR2 receptors recognize bacterial lipoproteins and lipotechoic acid from bacterial membranes, and they mediate the activation of transcription factors such as NF-κB and IFN-regulatory factors [19,32-34]. We found that at concentrations similar to those used in other studies (Table 1), all six TLR2 agonists tested inhibited fibrocyte differentiation (Figure 5). At day 5, the TLR2 agonist-treated cells appeared to be viable as determined by phase contrast microscopy (Figure 6). At day 5 after TLR2 agonist treatment, there was no significant difference in the number of viable PBMCs treated with or without TLR2 agonists (Figure 6B).

**Figure 7:**
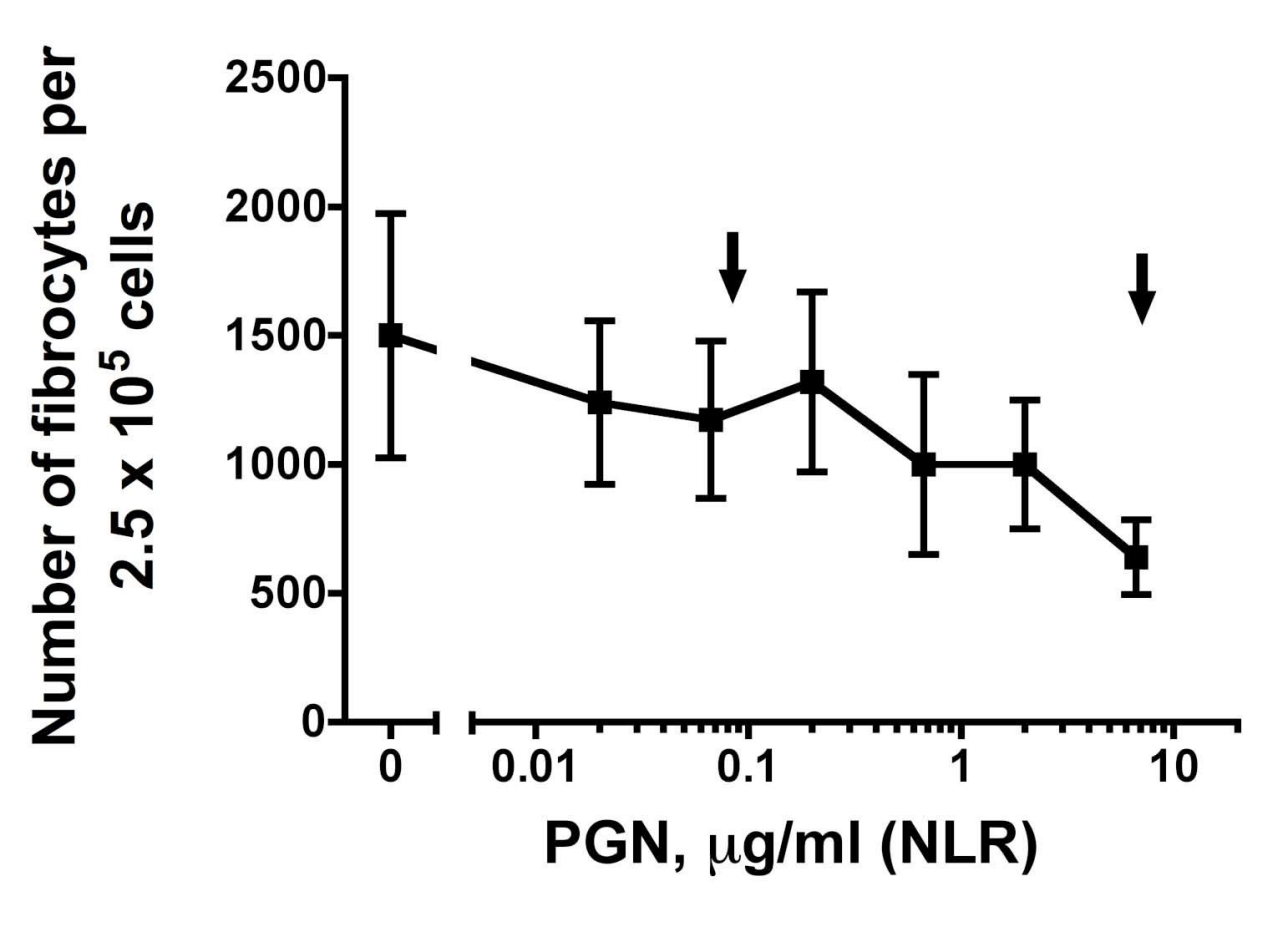
**The NLR agonist PGN does not affect fibrocyte differentiation**. Human PBMCs were cultured in different concentrations of PGN. After 5 days, the cells were air-dried, fixed and stained, and the number of fibrocytes was counted. The results are means ± SEM of fibrocytes per 2.5 × 10^5 ^PBMCs (n = 3 separate experiments). There was no statistically significant difference between the effect of any PGN concentration and control, according to ANOVA or *t*-test. Arrows indicate the concentrations of PGN used by other workers (Table 1).

Besides TLRs, there are also other pattern recognition receptors (PRRs) present in PBMCs that detect a variety of microbial molecules, including NLRs, that contain a NOD- and ligand-recognizing leucine-rich repeat [35]. NLRs are intracellular receptors that recognize a variety of microbial molecules. We investigated the role of PGN on fibrocyte differentiation, since this agonist appears to be a NLR agonist [36]. When added to PBMCs, PGN at concentrations from 0.01 to 6.7 μg/ml did not affect fibrocyte differentiation (Figure 7). Although not statistically significant, PGN increased the number of CD86- and MHC class II-positive cells (Figures 3B and 3C). Together, the data suggest that although PGN has no effect on fibrocyte differentiation when added to PBMCs, it still has some effect on cells.

### Some TLR2 agonists inhibit fibrocyte differentiation indirectly

**Figure 8:**
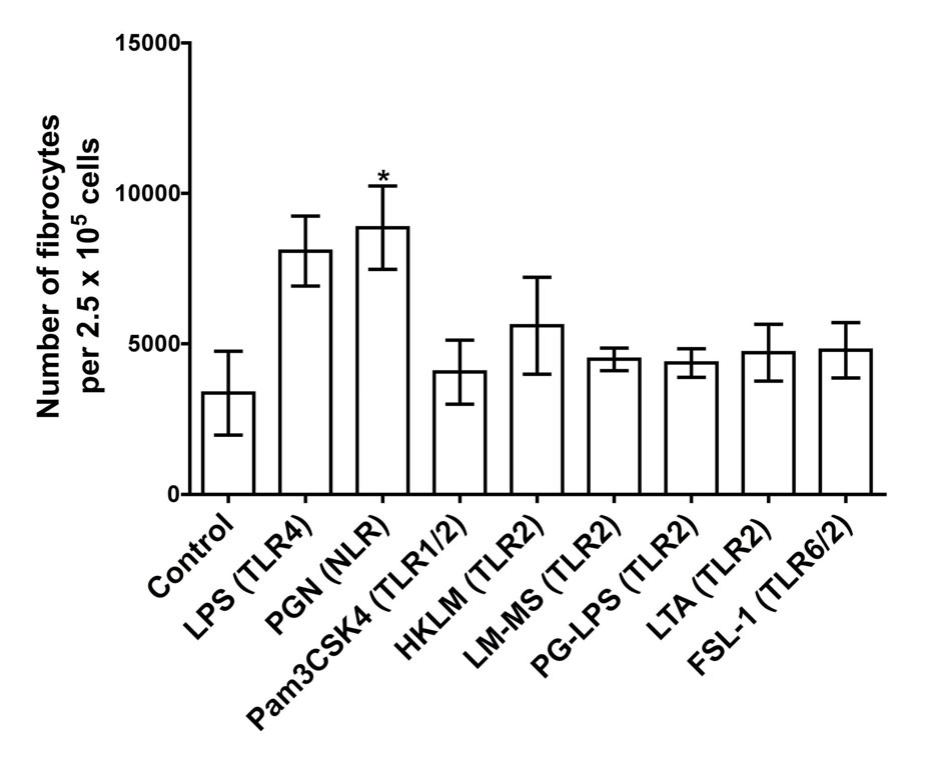
**TLR2 agonists do not inhibit the differentiation of purified monocytes to fibrocytes**. Human monocytes were cultured in SFM for 5 days in the presence of LPS (1 μg/ml), PGN (2 μg/ml), Pam3CSK4 (1 μg/ml), HKLM (1 × 10^8 ^cells/ml), LM-MS (1 μg/ml), PG-LPS (10 μg/ml), LTA (2 μg/ml) and FSL-1 (1 μg/ml). The results are means ± SEM of fibrocytes per 2.5 × 10^5 ^PBMCs (n = 4 separate experiments). *P < 0.05 compared to control (one-way ANOVA, Dunnett's test).

To determine whether TLR2 agonists act directly on human monocytes, we examined the effect of TLR2 agonists on purified monocytes. When TLR2 agonists were added to purified human monocytes, the TLR2 agonists did not inhibit the differentiation of monocytes to fibrocytes (Figure 8). This suggests that TLR2 agonists do not inhibit fibrocyte differentiation by acting directly on monocytes. Interestingly, the NLR agonist PGN potentiated the differentiation of monocytes to fibrocytes (Figure 8). Since PGN had little activity when added to PBMCs (Figure 7), PGN might act directly on monocytes to potentiate fibrocyte differentiation and this activity might be inhibited by the presence of the other cells in the PBMC population. TLR4 agonist LPS also potentiated the differentiation of monocytes to fibrocytes (Figure 8), though the increase was not significant by either ANOVA or *t*-test.

**Figure 9:**
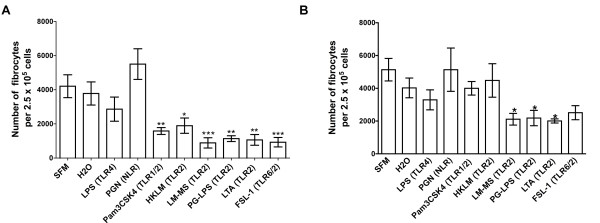
**Conditioned media from PBMCs incubated with LM-MS, PG-LPS and LTA inhibit fibrocyte differentiation**. **(A)**. Human PBMCs were cultured for 5 days in the presence of the TLR2 agonists Pam3CSK4, HKLM, LM-MS, PG-LPS, LTA and FSL-1, the TLR4 agonist LPS, and the NLR agonist PGN, using the same concentrations as in Figure 6. SFM was used as a buffer control, and LPS and PGN were used as negative controls, since these agonists do not inhibit fibrocyte differentiation. Conditioned media were removed on day 3 from duplicates of these wells. The results are means ± SEM of fibrocytes per 2.5 × 10^5 ^PBMCs (n = 5 separate experiments). *P < 0.05, **P < 0.01, and ***P < 0.001 compared to control (one-way ANOVA, Dunnett's test). **(B) **Human monocytes were cultured for 5 days in the presence of the conditioned media from day 3 PBMCs incubated with TLR2 agonists. The results are means ± SEM of fibrocytes per 2.5 × 10^5 ^PBMCs (n = 5 separate experiments). Conditioned media from PBMCs incubated with the TLR2 agonists LM-MS, PG-LPS and LTA significantly inhibited the differentiation of monocytes to fibrocytes. *P < 0.05 compared to control (one-way ANOVA, Dunnett's test).

Since TLR2 agonists do not act directly on monocytes, a possible explanation for the ability of TLR2 agonists to inhibit fibrocyte differentiation when added to PBMCs is that the TLR2 agonists cause some cells in the PBMC population, such as T, B or NK cells, to secrete a factor that inhibits the differentiation of monocytes to fibrocytes. To test this possibility, we incubated PBMCs for 3 days with TLR2 agonists, collected the conditioned media and then added this to monocytes from the same donor. As previously observed, when added to PBMCs, TLR2 agonists inhibited the differentiation of fibrocytes (Figure 9A). Conditioned media from PBMCs incubated with the TLR2 agonists lipomannans from *Mycobacterium smegmatis *(LM-MS), LPSs from *Porphyromonas gingivalis *(PG-LPS) and LTA for 3 days inhibited the differentiation of monocytes to fibrocytes (Figure 9B). This suggests that these TLR2 agonists cause some nonmonocyte cells in the PBMC population to increase the accumulation of fibrocyte-inhibiting factors or to decrease the accumulation of fibrocyte-promoting factors in the extracellular medium.

### Some TLR2 agonists cause PBMCs to secrete an unknown factor that inhibits fibrocyte differentiation

**Figure 10:**
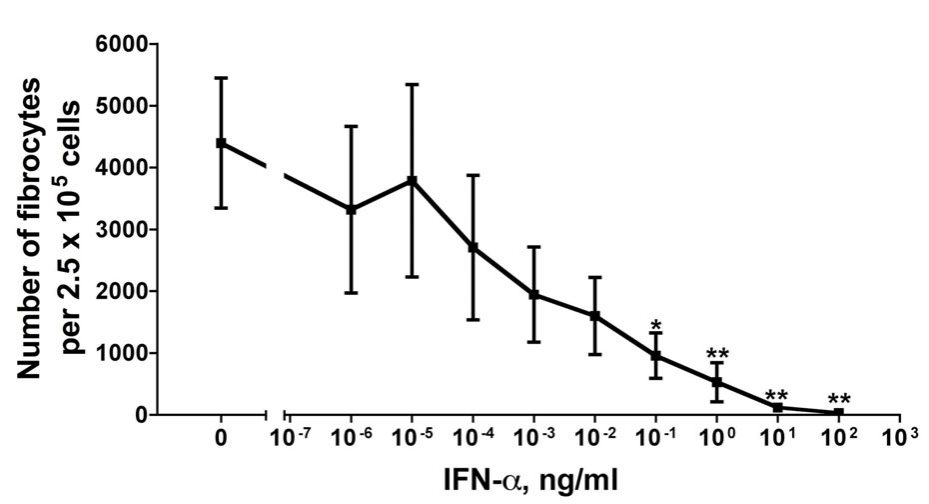
**Interferon (IFN)-α inhibits fibrocyte differentiation**. Human PBMCs were cultured in different concentrations of IFN-α. After 5 days, the cells were air-dried, fixed and stained, and the number of fibrocytes was counted. The results are means ± SEM of fibrocytes per 2.5 × 10^5 ^PBMCs (n = 3 separate experiments). *P < 0.05 and **P < 0.01 compared to no IFN-α control (one-way ANOVA, Dunnett's test).

**Figure 11:**
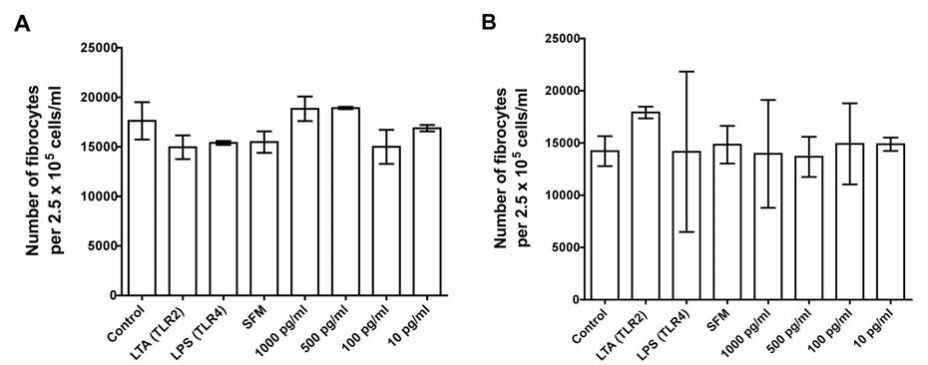
**Cytokine mixtures from SFM, LTA or LPS conditioned media had no effect on fibrocyte differentiation**. Human PBMCs were cultured in the absence of added cytokines (control) or in mixtures of cytokines (IL-6, TNF-α, granulocyte colony-stimulating factor (G-CSF), tumor growth factor (TGF)-β1, and IFN-γ) at the concentrations found in conditioned media from SFM-, LTA-, or LPS-treated PBMCs. Human PBMCs were also cultured in the presence of mixtures containing 1000 pg/ml, 500 pg/ml, 100 pg/ml, or 10 pg/ml of each of the five cytokines. After 5 days, the cells were dried, fixed and stained, and the number of fibrocytes was counted. The results are means ± SEM of fibrocytes per 2.5 × 10^5 ^PBMCs. **(A)**. Results from one donor. **(B) **Results from a second donor. For each donor, there was no significant difference between any cytokine mixture and the control (1-way ANOVA, Dunnett's test and *t*-test).

We previously found that 1 ng/ml or more IFN-γ, 0.5 μg/ml or more SAP or 10 μg/ml or more aggregated IgG directly inhibits monocytes from differentiating into fibrocytes and that 1 ng/ml or more IL-12 indirectly inhibits fibrocyte differentiation [5-7]. In cultures of PBMCs containing fetal bovine serum, IFN-α inhibits fibrocyte differentiation [13]. In our serum-free culture system, 0.1 ng/ml or more IFN-α also inhibited fibrocyte differentiation (Figure 10). To determine whether one of these inhibitory factors is the antifibrocyte factor present in conditioned media from PBMCs incubated with the TLR2 agonists LM-MS, PG-LPS and LTA, we examined the levels of IFN-α, IFN-γ, IL-12, SAP and IgG in the conditioned media. Conditioned media from PBMCs incubated with TLR2 agonists as well as the TLR4 agonist LPS did not show any detectable levels of IL-12 or SAP, with a detection limit of 0.1 ng/ml for both factors (data not shown). These conditioned media also had less than 0.01 ng/ml IFN-γ (Table 2 and data not shown). The conditioned media also did not show any detectable levels of either IFN-α or IgG, with a detection limit of 1 pg/ml IFN-α and 1 μg/ml IgG (data not shown). We further analyzed day 3 conditioned media from PBMCs incubated with Pam3CSK4, PG-LPS, LTA, PGN and LPS for the presence of Th1/Th2/Th17 cytokines. There were no detectable levels of IL-2, IL-4, IL-5, IL-10, IL-12, IL-13 or IL-17A with a detection level of approximately 15 pg/ml for each of the cytokines. We detected IL-6, TNF-α, IFN-γ, G-CSF and TGF-β1 in conditioned media from LTA (TLR2 agonist)-treated PBMCs, LPS (TLR4 agonist)-treated PBMCs, and control PBMCs (Table 2). We then analyzed the effect of cocktails of these five cytokines on fibrocyte differentiation. Cytokine cocktails corresponding to the amount of the five cytokines found in LTA-treated conditioned media, LPS-treated conditioned media or control conditioned media did not have any significant effect on fibrocyte differentiation (Figure 11). In addition, cocktails containing higher levels of the five cytokines had no significant effect on fibrocyte differentiation (Figure 11). These observations indicate that the LTA-induced factor in the PBMC conditioned media that inhibits fibrocyte differentiation is not one of the five cytokines that we found in LTA-treated conditioned media and thus appears to be a novel factor.

**Table 2:** Conditioned media from LTA-treated PBMC, LPS-treated PBMC, and control conditioned media contain low levels of IL-6, TNF-α, IFN-γ, G-CSF and TGF-β1

Cytokine	Concentration in control CM (pg/ml)	Concentration in LPS-treated CM (pg/ml)	Concentration in LTA-treated CM (pg/ml)
IL-6	4 ± 12	530 ± 250	750 ± 250
TNF-α	4.6 ± 1.6	29 ± 13	33 ± 6
IFN-γ	1.0 ± 0.5	2.4 ± 0.9	4.5 ± 3.5
G-CSF	2.5 ± 0	17 ± 0	9.3 ± 1.8
TGF-β1	13 ± 1.8	3.5 ± 0	4.5 ± 0

The above five cytokines were detected in conditioned media (CM) from LTA-treated PBMCs, LPS-treated PBMCs and control PBMCs. This assay measures total tumor growth factor (TGF)-β1 as opposed to active TGF-β1. The results are means ± SEM of cytokine concentration (n = 2 separate experiments).

## Discussion

We found that a variety of TLR2 agonists inhibit fibrocyte differentiation. However, the TLR2 agonists do not directly inhibit the differentiation of monocytes to fibrocytes, but rather cause some other cell type in the PBMC population to inhibit monocytes from differentiating into fibrocytes. There are five known factors (IFN-α, IFN-γ, IL-12, SAP and aggregated IgG) which inhibit fibrocyte differentiation [5-7,13]. Our data suggest that PBMCs incubated with some TLR2 agonists secrete an unknown sixth factor that inhibits fibrocyte differentiation.

The TLR2 agonist LTA induces PBMCs to increase the extracellular accumulation of IL-6, TNF-α, IFN-γ and G-CSF and to decrease the extracellular accumulation of TGF-β1. Both TLR2 agonists and TLR4 agonist induce primary adrenocortical cells to secrete IL-6 [37,38]. However, we previously found that IL-6 has no effect on fibrocyte differentiation [7]. Like the above five factors, this combination of cytokines at the concentrations found in the conditioned media, or at higher concentrations, was unable to inhibit fibrocyte differentiation and is thus not the factor in LTA-stimulated conditioned medium that inhibits fibrocyte differentiation.

Monocytes express TLR1-TLR9, but not TLR10 [39,40]. They have a high expression of TLR1, TLR2 and TLR4; intermediate expression of TLR 5, TLR6 and TLR8; and low expression of TLR7 and TLR9 [39,40]. Once activated, TLR agonists trigger downstream signaling events by one of four adaptor molecules: MyD88, MyD88-like adaptor protein (Mal), Toll/Interleukin-1 receptor (TIR) domain-containing adaptor protein-inducing IFN-β (TRIF) or TRIF-related adaptor molecule (TRAM) [41-45]. MyD88 activates mitogen-activated protein kinases (MAPKs) through IL-1R-associated kinase (IRAK) and *tumor necrosis factor receptor *(TNFR)-associated factor 6 (TRAF-6) [41,45]. The signaling eventually causes the translocation of transcription factors such as NF-κB and activator protein 1 (AP-1), which then induces the production of inflammatory cytokines such as TNF-α, IL-6, IL-1β and IL-12 [41]. TLR3 does not require the MyD88 pathway; instead it induces the production of IFN-β via TRIF [41,46]. TLR4 can cause NF-κB activation by either a MyD88-dependent pathway or a MyD88-independent pathway [42]. TLR4 activates a MyD88-independent pathway via TRIF, which complexes with TRAM and leads to NF-κB activation [42]. Since none of the TLR agonists appeared to directly inhibit the differentiation of monocytes to fibrocytes, our data suggest that none of the above signal transduction pathways in monocytes affects their ability to differentiate into fibrocytes.

TLR2 recognizes various bacterial components such as bacterial lipoproteins and LTA [41,47]. PBMCs incubated with the TLR2 agonists LM-MS, PG-LPS and LTA secrete an unknown factor that inhibits fibrocyte differentiation. However, for unknown reasons, the TLR2 agonists Pam3CSK4 (a synthetic triacylated lipopeptide), heat-killed *Listeria monocytogenes *(HKLM) and FSL-1 (a synthetic lipoprotein) do not appear to cause other cells in the PBMC population to secrete factors that inhibit fibrocyte differentiation. This suggests that these agonists indirectly inhibit fibrocyte differentiation by either cell-cell contact or a labile secreted factor.

We previously found that aggregated IgG inhibits fibrocyte differentiation [6]. We hypothesized that since aggregated IgG (that is, IgG bound to something such as a bacterium) signifies the presence of bacterial infection, and since closing an infected wound and thus forming an anaerobic pocket can be detrimental, the inhibition of fibrocyte differentiation by aggregated IgG might have evolved to prevent closure of septic wounds by fibrocytes. An interesting possibility is that the indirect inhibition of fibrocyte differentiation by TLR2 agonists allows other cells in a wound environment to check for the presence of bacteria, and if bacteria are present, to relay this information to monocytes and prevent fibrocyte differentiation and the possible closure of an infected wound.

## Conflict of interest

The authors declare that they have no competing interests.

## Authors' contributions

ASM conceived and designed the experiments, performed the experiments, analyzed the data and wrote the paper. DP conceived and designed the experiments, performed the experiments and edited the manuscript. RHG conceived and designed the experiments, analyzed the data and edited the manuscript. All authors read and approved the final manuscript.
